# Pulmonary endemic mycoses

**DOI:** 10.1016/j.clinme.2024.100014

**Published:** 2024-01-23

**Authors:** Michael J. Wilson, Irasha Harding, Andrew M. Borman, Elizabeth Johnson, Robert Miller

**Affiliations:** a SpR in infectious diseases and microbiology, University Hospitals Bristol and Weston NHS Foundation Trust, Bristol, UK; b consultant microbiologist, National Infection Service, Bristol, UK; c deputy director, UKHSA National Mycology Reference Laboratory, Bristol, UK; d honorary professor of medical mycology, University of Exeter, Exeter, UK; e director, UKHSA National Mycology Reference Laboratory, Bristol, UK; f Associate Professor of Clinical Infection, Institute for Global Health, University College London

**Keywords:** Endemic mycoses, Pulmonary, Infection, Fungi, Immunocompromised

## Abstract

While rare, the likelihood of encountering a case of a pulmonary endemic mycosis (PEM) in the UK is increasing. Diagnosis may be challenging, often leading to considerable delay to appropriate treatment. Clinical suspicion must be present for respiratory disease, particularly in the immunocompromised or in those not responding to empiric treatment approaches, and an extended travel history should be obtained. This article summarises the epidemiology of PEM, key clinical features, diagnostic strategies and management.

## Introduction

The endemic mycoses are a group of diseases caused by dimorphic fungi capable of infecting humans. Dimorphic relates to their two physical states: a mould in colder environmental temperatures and a yeast, or a spherule in the case of *Coccidioides*, in warmer mammalian temperatures. They are termed ‘endemic’ because infections have traditionally been linked to specific geographic areas. However, wider distributions are now being observed, perhaps related to improved recognition and diagnostics, or perhaps due to spread within an increasingly connected and warming world;^1^ the relative ease of international travel has also amplified the potential for exposure, and with broader use of immunosuppression there is a growing population who are more vulnerable. Consequently, there is a need for clinicians outside endemic countries to be aware of pulmonary endemic mycosis (PEM).

Histoplasmosis, coccidioidomycosis, blastomycosis, paracoccidioidomycosis, talaromycosis and emergomycosis are all examples of a PEM. Inhalation of fungal particles is the predominant route of infection, leading to respiratory disease as a common feature, with or without subsequent systemic dissemination. Diagnosis can be challenging, both clinically and in the laboratory: presenting symptoms may have a broad differential, with acute disease manifesting as an influenza-like illness or community-acquired pneumonia (CAP), and chronic disease mimicking tuberculosis (TB) or cancer; imaging is non-specific (Table 1);^2^, ^3^, ^4^ and laboratory diagnostics (Table 2), comprising direct fungal microscopy, culture, serological testing and PCR, usually require access to a reference laboratory and may take several weeks (4 weeks for fungal culture can be necessary for some PEM).^4^^,^^5^ Often, multiple trials of futile antibiotics will have been administered in the interim, something to be avoided in our world of increasing antimicrobial resistance.

**Table 1:** Imaging – an overview of possible chest imaging appearances in endemic mycoses.

Radiological characteristics	Histoplasmosis	Coccidioidomycosis	Blastomycosis	Paracoccidioidomycosis	Talaromycosis
Consolidation (single or multiple lobes)		✓	✓	✓	
Pulmonary infiltrates	✓	✓	✓		✓
Nodules	✓	✓	✓	✓	✓
Miliary pattern	✓	✓	✓		✓
Cavities	✓	✓	✓	✓	✓
Mediastinal lymphadenopathy	✓	✓		✓	✓
Pleural effusions	✓	✓	✓	✓ (rare)	✓

**Table 2:** Laboratory diagnostics.

Diagnostic method	Sample types	Notes
**Fungal microscopy**	Sputum, BAL fluid, tissue, bone marrow	• Varying sensitivity dependent on quantity of fungus in sample and experience of microscopist • Use of calcofluor staining enhances detection, or giemsa-staining of bone-marrow for *Histoplasma*
**Fungal culture**	Sputum, BAL fluid, tissue, blood, bone marrow	• Gives definitive diagnosis • Fungal growth can be very slow, 4 weeks in some cases; *Coccidioides* is faster, growing in a few days
**Antibody detection**	Serum	• Antibody development may take weeks • Less useful in early infection or in the immunocompromised • Cross-reactivity of antibodies reduces specificity
**Antigen detection**	Serum, BAL fluid, urine	• Beta-D glucan – a non-specific fungal biomarker, may be raised in *Coccidioides, Histoplasma, Talaromyces* and *Paracoccidioides* infections, less useful with *Blastomyces*, which produces lower levels • Antigen testing (including urinary) is available for *Blastomyces* and *Histoplasma* • An assay for *Talaromyces* is in use in China
**NAAT**	BAL fluid, tissue	• Pan-fungal PCR may be useful • Specific PCR tests are currently more limited to research

BAL = bronchoalveolar lavage, NAAT = nucleic acid amplification test, PCR = polymerase chain reaction.

While improvements in rapid laboratory diagnostics are awaited, early clinical suspicion is needed to expedite diagnosis and allow initiation of relevant treatment. Travel history, including historic travel and previous countries of residence, is essential. Location and activities can provide good clues and soil/mud/dust disturbance may be the initial exposure. An incubation period of a few weeks is typical in acute infection, but chronic disease can resurface months, years or even decades later, as is the case with *Paracoccidioides*. In non-endemic countries PEM is more commonly seen in the immunocompromised, resulting from travel-related infection or immunosuppression-induced reactivation. Emergomycosis is an emerging disease predominantly identified in the immunocompromised.^5^ Further details on other PEM are summarised below and in Table 3.

**Table 3:** Brief overview of pulmonary endemic mycoses.

	Histoplasmosis	Coccidioidomycosis	Blastomycosis	Paracoccidioidomycosis	Talaromycosis
Major endemic areas*	Southeastern Canada, eastern USA, Central and South America, sub-Saharan Africa, South and East Asia	Southwestern USA, Mexico	Central and eastern North America, Africa	Central and South America, predominantly Brazil	South and Southeast Asia
Risk factors	Exposure to soil containing bird or bat guano	Dry soil/dust disruption	Disrupted vegetation/ rotting wood near freshwater	Soil exposure	Exposure to soil in rainy season Immunocompromise, particularly advanced HIV
Clinical manifestations	Acute: usually self-limiting influenza-like-illness Chronic: pulmonary disease mimicking TB	Community acquired pneumonia	Indolent pulmonary disease Extrapulmonary disease	Acute: lymphadenopathy and fever Chronic: pulmonary involvement in majority, skin lesions	Umbilicated skin lesions Disseminated disease
Key diagnostic tests	Microscopy and culture Urinary antigen test Beta-D glucan Galactomannan	Microscopy and culture Serology	Microscopy and culture Serology	Microscopy and culture Serology	Microscopy and culture Beta-D glucan Galactomannan
Treatment	May not be required for primary pulmonary disease If more severe/ disseminated use itraconazole or L-AmB	Not required for primary pulmonary disease If immunocompromised or if more severe/ disseminated disease use itraconazole, fluconazole (CNS disease) or L-AmB	Itraconazole or L-AmB	Itraconazole or L-AmB	L-AmB then itraconazole
Cases referred to the MRL yearly**	18–24	3–6	1–2	1–2	1–2

CNS = central nervous system, HIV = human immunodeficiency virus, L-AmB = liposomal amphotericin B, MRL = Mycology Reference Laboratory (UK), TB = tuberculosis

⁎ = non-exhaustive list

⁎⁎ = likely an underestimation of total UK cases

## Laboratory diagnostics

Given the difficulty in diagnosis, multiple testing approaches should be employed simultaneously (Table 2). Extended fungal culture must be requested with relevant clinical information to prevent samples being discarded after routine durations. Referral to reference laboratories is recommended. Samples should be processed in biosafety level 3 laboratories due to biohazard the risk to laboratory staff.

## Histoplasmosis

**Fig 1:**
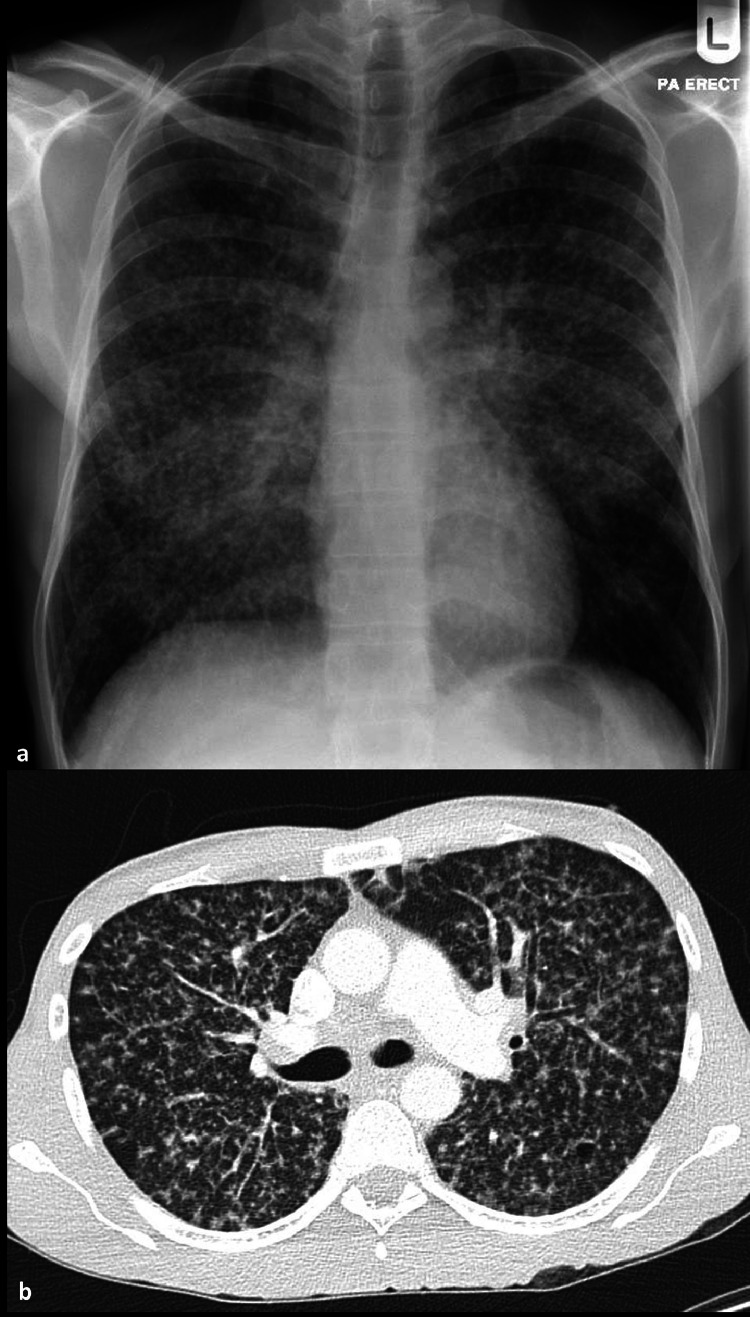
**Radiological images from a patient with pulmonary histoplasmosis.** (a) Chest X-Ray and (b) computed tomography. The radiological appearance mimicks that of miliary tuberculosis.

*Histoplasma* is now understood to be present across much of the globe, including eastern USA, southeastern Canada, Central and South America, sub-Saharan Africa, South and East Asia and the east coast of Australia.^1^ Particularly found in soil containing bird and bat guano. Lifetime exposure rates can be nearly universal (90%) for those living in hyperendemic areas, such as the Mississippi and Ohio river basins in the USA and parts of Brazil.^6^ Travel to Latin America has been associated with high (20%) exposure rates.^7^ Acute pulmonary histoplasmosis is usually asymptomatic or a self-limiting influenza-like illness starting 3–21 days after exposure.^3^ High inoculum load may lead to a more severe acute illness and outbreaks have been linked to cave exploration.^8^ Chronic pulmonary histoplasmosis is rarer, mostly affecting those with underlying lung disease, and can appear similar to TB with high morbidity if untreated (Fig 1).^9^ Infection in the immunocompromised is often disseminated and can be life-threatening. Histoplasmosis is a major cause of morbidity and mortality in advanced HIV. A lateral flow urinary antigen test is commercially available for diagnosis. Treatment of severe disease is with liposomal amphotericin B (L-AmB) followed by itraconazole. Itraconazole is also used for less severe disease. Treatment duration should be at least 6 months, rising to at least 1 year if immunocompromised where longer term maintenance therapy may also be required.^5^

## Coccidioidomycosis

**Fig 2:**
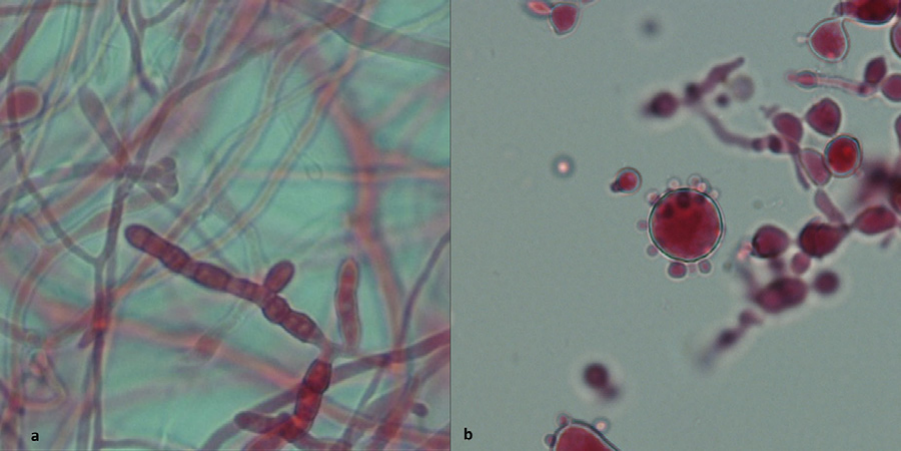
**The organisms causing occidioidomycosis and paracoccidioidomycosis.** (a) *Coccidioides immitis* in the environmental mould form. X 400 magnification, lactofuchsin stain. Thick-walled, barrel-shaped arthroconidia with thin-walled empty cells between, forming in mature hyphae. Infectious arthroconidia are released when the thin-walled empty cells rupture. (b) *Paracoccidioides brasiliensis* in the yeast phase. X 400 magnification, lactofuchsin stain. Characteristic ship's wheel appearance of a mother cell with multiple synchronously budding daughter cells.

This is predominantly found in southwestern USA and Mexico, and also in arid regions of South America. There are an estimated 350,000 annual cases in the USA, with the majority in southern Arizona and the San Joaquin valley region in California, where coccidioidomycosis may account for a high proportion (15–29%) of all CAP diagnoses.^10^, ^11^, ^12^ *Coccidioides* can survive in dry areas and infection is typically acquired when arthroconidia become airborne following soil disruption, there can be a very low infectious dose (Fig 2). Outbreaks have been linked to dust storms, military training exercises and construction.^13^ Incidence is higher in summer months. Many (60%) have asymptomatic infection, with others presenting with a picture of CAP developing 1–3 weeks after exposure. Most are diagnosed by serology. Unlike the other endemic mycoses, pulmonary coccidioidomycosis often requires no treatment and most patients will improve spontaneously. If the patient is immunocompromised, or there is evidence of extrapulmonary disease, treatment is indicated. Fluconazole or itraconazole are used for milder infections, L-AmB for severe or disseminated disease.^5^^,^^14^

## Blastomycosis

Endemic to eastern North America, multiple cases have also been reported in Africa, the Middle East and India. The environmental niche for *Blastomyces* is damp vegetation-rich soil, near freshwater. Infection is via inhalation of spores.^1^^,^^5^ The commonest presentation is with pulmonary involvement, often with an indolent picture, but it can mimic CAP or even progress to acute respiratory distress syndrome. 50% of infections may be asymptomatic. Of those with symptomatic infection, roughly a third may develop extrapulmonary disease including skin, bone, genitourinary and central nervous systems, mimicking metastatic malignancy. Treatment of mild to moderate disease is with itraconazole. In severe disease, or with central nervous system involvement, L-AmB should be used. The duration is 6–12 months and mortality can reach 4–22%.^15^

## Paracoccidioidomycosis

This is endemic to South America, Central America and southern Mexico but mainly in Brazil. Extremely high population exposure has been documented in endemic regions, with 50–75% positivity on skin testing. *Paracoccidioides* is a soil-dwelling fungus, although rarely identified in environmental samples. Very slow-growing by culture, which may take up to 30 days, direct microscopy can be more useful with identification of the characteristic yeast cell (Fig 2). Serological testing leads to the bulk of diagnoses. Clinical disease is broadly divided into two groups: acute/subacute and chronic. The acute/subacute form is seen in children, adolescents and young adults, with a picture of lymphadenopathy, fever and weight loss. Symptoms last around 2 months. The chronic form is felt to represent reactivation or reinfection and is seen in adults over 30 with a marked male-to-female ratio of 20:1.^5^ Pulmonary involvement is usual, with chronic symptoms lasting over 6 months that can mimic TB. Paracoccidioidomycosis is often a multi-organ disease and skin lesions are frequently the trigger to seek medical attention. All confirmed cases should be treated; mild disease is treated with itraconazole while L-AmB is used for severe disease or in the immunocompromised. There can be a high rate of relapse and subsequent itraconazole or co-trimoxazole should be given as maintenance treatment. Duration is a minimum of 6 months and can be years if co-trimoxazole is chosen as maintenance therapy.^16^

## Talaromycosis

*Talaromyces marneffei* (formerly *Penicillium marneffei*) is endemic through South and Southeast Asia. Infection has been associated with exposure to soil in the rainy season, with infection via inhalation prior to haematogenous spread and dissemination.^4^ Predominantly an infection of the immunocompromised and a major cause of opportunistic infection in HIV, a dramatic rise in incidence was observed with rising HIV rates in the 1990s.^17^ Skin lesions are a major feature in HIV positive individuals with raised, umbilicated lesions; however, this molluscum-like rash is not a specific finding of talaromycosis and similar lesions may be seen in histoplasmosis and cryptococcosis. Cases of isolated pulmonary disease have also been described. Antigen detection (Mp1p) has shown good promise for rapid and accurate diagnosis, particularly in those with high disease burden, and is approved for use in China. Talaromycosis can carry a high mortality of 25% even with treatment, increasing significantly with delayed diagnosis.^18^^,^^19^ L-AmB is the preferred induction treatment for 10-14 days, followed by itraconazole consolidation therapy for 10 weeks and maintenance therapy thereafter.^5^

## Conclusion

The pulmonary endemic mycoses are infections primarily acquired through inhalation of airborne fungal particles disturbed from their environment. While previously felt to be very localised geographically, there is increasing recognition of a wider geographic distribution. Combined with an increasing population at risk of exposure through travel and immunosuppression, it is important to have an awareness of these diseases so that investigations can commence early to reduce delays to diagnosis and treatment.

**Key points**

• The pulmonary endemic mycoses may become more prevalent in a warming and connected world. Histoplasmosis is by far the most prevalent imported dimorphic mycosis in the UK today, with more than 20 cases diagnosed annually.
• Clinical suspicion is essential for expedient diagnosis, but a relevant travel history is imperative to identify the most useful tests to perform.
• Diagnosis can be difficult especially in immunocompromised patients: samples should be sent for multiple laboratory diagnostic tests simultaneously to maximise detection. All samples for microscopy, culture and PCR should be handled under biosafety level 3 containment.
• If clinically indicated, the mainstay of treatment for mild-moderate disease with pulmonary endemic mycoses is itraconazole, apart from *Coccidioides* in which case fluconazole is used.
• Severe disease is treated with amphotericin B, usually the liposomal form.

Alt-text: Unlabelled box
